# Patient Adherence to Hemoglobin A_1c_ Testing Recommendations in Telemedicine and In-Office Cohorts During COVID-19

**DOI:** 10.1001/jamanetworkopen.2021.27779

**Published:** 2021-09-30

**Authors:** Derek Baughman, Areeba Zain, Abdul Waheed

**Affiliations:** 1 WellSpan Good Samaritan Hospital Family Medicine Residency Program, Lebanon, Pennsylvania

## Abstract

This cohort study assesses patient adherence to diabetes screening using hemoglobin A_1c_ level at in-person vs telemedicine encounters during the COVID-19 pandemic in the US.

## Introduction

Telemedicine can reduce unnecessary health care utilization, disease management and travel costs, and the financial impact of patient no-shows.^1,2^ Telemedicine may also improve the patient-clinician experience, wait times, medication adherence, and overall satisfaction.^3^ However, telemedicine quality of care is not well studied. National organizations recommend universal screening for prediabetes and diabetes^4^; in this study, we used a national reporting measure to compare adherence to diabetes screening between in-person and telemedicine encounters during COVID-19.

## Methods

In a retrospective cohort analysis that was determined to be exempt by the WellSpan Health Institutional Review Board, we compared the percentage of encounters in which recommended hemoglobin A_1c_ (HbA_1c_) testing^5^ was completed within the 6 months after an in-office or telemedicine encounter in a large health system. Testing could be point-of-care office testing or laboratory testing. Encounters were included if it was a visit with any specialty outpatient clinician between January 1, 2020, and January 1, 2021, and an HbA_1c_ laboratory test was ordered at that encounter. We used Epic’s Slicer Dicer to compare the adherence percentages in patients with and without diabetes and family medicine (FM) vs all other outpatient specialties. We compared rates to a 3-year historical office-only pre–COVID-19 average. We stratified encounters by electronic medical record–reported race, age, sex, ethnicity, social determinants of health, and overall risk score (eMethods table in the Supplement). “N − 1” χ^2^ tests detected statistical significance of adherence rates between subgroups (*P* < .05). This study followed the Strengthening the Reporting of Observational Studies in Epidemiology (STROBE) reporting guideline.

## Results

Of the 521 234 outpatient encounters during the study period, 63 722 met inclusion criteria. Among included participants, the mean (SD) age was 62 (16) years, 27 667 (51.5%) were female, 53 973 (84.7%) were White, and patient overall risk score was low. Most office and telemedicine encounters were with FM clinicians (Table). Rates of office-only HbA_1c_ laboratory test completion decreased from the 3-year historical prepandemic average of 74.2% to 60.2% during the pandemic. There was a 4.2% higher laboratory test adherence for office encounters compared with telemedicine visits during the pandemic—for all-specialties, all patients (60.2% vs 56.0%, *P* < .001). Patients with diabetes had a 7.9% laboratory test higher adherence with office visits compared to telemedicine visits (68.1% vs 60.2%, *P* < .001); there was no difference between office and telemedicine visits in laboratory test adherence rates for patients without diabetes (0.4%, *P* = .64). There were no differences in our other stratified analysis.

**Table.** HbA_1c_ Laboratory Test Adherence Rates During COVID-19 in Patients With and Without Diabetes Compared by Encounter Type and Specialty Management^a^

	Telemedicine		Office		Telemedicine + office		Adherence rate differences (office vs telemedicine)	
	HbA_1c_ ordered, No.	Adherent in 6 mo, No. (%)	HbA_1c_ ordered, No.	Adherent in 6 mo, No. (%)	HbA_1c_ ordered, No.	Adherent in 6 mo, No. (%)	Difference, %	*P* value
All specialties								
With diabetes^b^	4822	2904 (60.2)	29 029	19 775 (68.1)	33 851	22 679 (67.0)	7.9	<.001
Without diabetes	4028	2056 (51.0)	25 843	13 278 (51.4)	29 871	15 334 (51.3)	0.4	.64
Total	8850	4960 (56.0)	54 872	33 053 (60.2)	63 722	38 013 (59.7)	4.2	<.001
Family medicine (only)								
With diabetes^b^	2760	1675 (60.7)	19 675	12 954 (65.8)	22 435	14 629 (65.2)	5.1	<.001
Without diabetes	2141	1087 (50.8)	17 332	8828 (50.9)	19 473	9915 (50.9)	0.1	.93
Total	4901	2762 (56.4)	37 007	21 782 (58.9)	41 908	24 544 (58.6)	2.5	.01
Other specialties (excluding FM)								
With diabetes^b^	2062	1229 (59.6)	9354	6821 (72.9)	11 416	8050 (70.5)	13.3	<.001
Without diabetes	1887	969 (51.4)	8511	4450 (52.3)	10 398	5419 (52.1)	1.8	.16
Total	3949	2198 (55.7)	17 865	11 271 (63.1)	21 814	13 469 (61.7)	7.4	<.001

Abbreviations: FM, family medicine; HbA_1c_, hemoglobin A_1c_.

^a^ Subgroup analysis of HbA_1c_ adherence rate differences between telemedicine vs office encounters calculated with “N − 1” χ^2^ tests (proportion differences with *P* value of .05).

^b^ Diabetes refers to type 2 diabetes. Electronic medical record data were selected for diabetes *International Statistical Classification of Diseases, Tenth Revision, Clinical Modification (ICD-10-CM) *code group: ICD-10-CM E11.

## Discussion

Overall, we found statistically significantly higher rates of HbA_1c_ adherence in office visits compared with telemedicine encounters during the COVID-19 pandemic. The difference was smaller for FM-only encounters, which is meaningful because FM physicians completed nearly double the encounters and HbA_1c_ ordering compared with all other specialties combined.

We also found moderately higher rates of HbA_1c_ adherence for diabetic patients in office vs telemedicine encounters. This may be due to diabetic patients understanding the value of HbA_1c_ testing, that the offices they visited had on-site laboratory tests or point-of-care testing, or the culture of specialty care (where high demand to see clinicians might foster an environment of health literacy). Regardless, the use of National Quality Forum’s standardized measure suggests that quality of diabetes care is close to that of in-office visits.^5^

Our most notable finding was no difference in adherence rates for patients without diabetes who had telemedicine encounters. This demonstrates the benefit of telemedicine in preventive care—comparable quality with lower cost.^2^ Similar to recent literature,^6^ during the pandemic, telemedicine met a care demand in our study; but moving forward, telemedicine may be a valuable care venue, especially in primary care.

Our study is limited because it was conducted in a single health system and had no pre–COVID-19 control for telemedicine (because of miniscule pre–COVID-19 numbers) and it is not clear that the differences we measured were clinically meaningful. Higher-level studies involving randomization to encounter type would provide important evidence for quality of telemedicine care.

## Notes

Supplement. **eAppendix. **Supplementary Materials
