# Supplementary material for: Patient Adherence to Hemoglobin A1c Testing Recommendations in Telemedicine and In-Office Cohorts During COVID-19
Source: JAMA Netw Open. 2021 Sep 30;4(9):e2127779. doi: 10.1001/jamanetworkopen.2021.27779 (PMC8485170; doi:10.1001/jamanetworkopen.2021.27779)
Supplement: Supplement. — eAppendix. Supplementary Materials [file jamanetwopen-e2127779-s001.pdf]

Supplemental Online Content

Baughman D, Zain A, Waheed A. Patient adherence to hemoglobin A_{1c} testing recommendations in telemedicine and in-office cohorts during COVID-19. *JAMA Netw Open*. 2021;4(9):e2127779. doi:10.1001/jamanetworkopen.2021.27779

eAppendix. Supplementary Materials

This supplemental material has been provided by the authors to give readers additional information about their work.

© 2021 Baughman D et al. *JAMA Network Open.*

eAppendix. Supplementary Materials

Controlling for sampling bias: the importance of avoiding timeframe selection during COVID-19
As desirable, larger national data set such as Medicare claims were difficult to acquire short term (thus precluding timely snapshots of quality), this introduced a sampling bias: a single integrated healthcare system comprising 20,000 employees, 8 hospitals and 2,600 clinicians in >200 outpatient care sites across South-Central Pennsylvania. This bias was addressed with timeframe stratification by encounter type pre-pandemic (1/1/17-1/1/20) to compare volume and adherence rates intra-pandemic (1/1/20-1/1/21) (figure 1). Due to insufficient encounter numbers to establish a telemedicine comparison group (only 17 total telemedicine encounters from 2019-2020 and none prior to that), we observed the trend in office-only volume (pre-pandemic into the pandemic timeframe) to facilitate context for comparing intra-pandemic telemedicine volume and adherence rates to the office encounter equivalents (reasonable considering similar data trends in other recent studies on volume of primary care utilization[1]). Importantly, unlike recent studies[2], time frame selection was avoided in our data set since the NQF states that this can impair validity and reliability of results[3,4]. To control for additional COVID-19 confounding, office only encounter data was compared to a 3-year historical adherence rate (figure 1).

Figure 1: Office-only encounters comparing volume and HbA1c adherence from 2019-2020, overlaid 2020 telemedicine volume and adherence (all specialties)

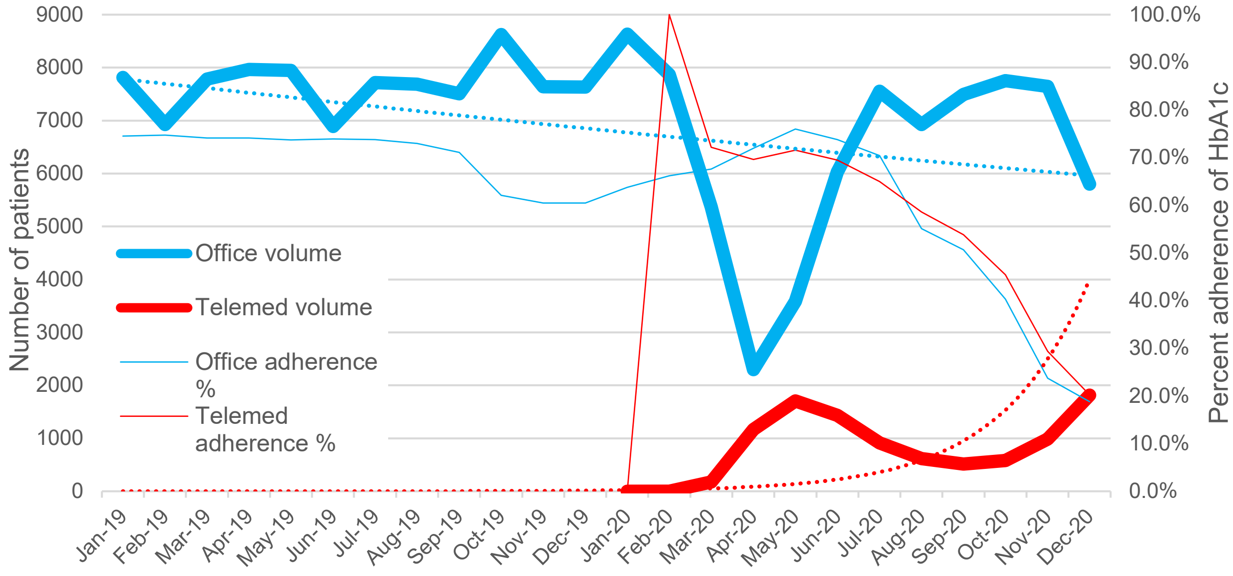

Controlling for COVID-19 confounding: average overall adult risk score

In addition to age, ethnicity, sex and SDOH, an "overall adult risk score" was used as an indicator of health risk based on: (1) number of diagnoses (*asthma, coronary artery disease, congestive heart failure, chronic obstructive pulmonary disease, chronic kidney disease, diabetes and hypertension*), (2) SDOH (*age, depression risk, tobacco use, financial resource strain risk, food insecurity risk, transportation needs risk, physical inactivity risk, intimate partner violence risk, social isolation risk, and alcohol risk score*), (3) healthcare utilization (*recent emergency room visits or hospital admissions*). Additional SDOH were stratified by race to specifically evaluate housing and financial strain. The "average overall adult risk score" although not a validated scoring system (as it was designed by the WellSpan health system) was plausibly superior to other less granular standardized scoring systems[5,6] because in addition to comorbid conditions, it accounted for SDOH and healthcare utilization (table 1).

© 2021 Baughman D et al. *JAMA Network Open.*

Table 1: Stratification of population by demographic data and encounter type												
	**Physical demographics**				**SDOH:** Avg # places lived last year		**SDOH:** Financial resource strain		**Avg overall adult risk score***		**6mo HbA1c adherence**	
	**Race**	**Age (avg)**	**Male**	**Female**	Telemed	Office	Telemed	Office	Telemed	Office	Telemed	Office
**White**	84.7%	63	49.0%	51.0%	1	1	9.5%	10.0%	5	6	58.3%	62.4%
**Other**	9.3%	52	46.5%	53.5%	1	1	10.0%	11.0%	6	5	60.3%	63.5%
**Black/African American**	4.8%	54	43.4%	56.6%	1	**2**	11.2%	14.7%	6	6	61.4%	61.8%
**Asian**	1.1%	58	46.0%	54.0%	1	1	4.9%	5.0%	4	4	**66.3%**	62.4%
**Totals**		62	48.5%	51.5%	1	1	10.2%	9.6%	5	6		
* Average overall adult risk score based on WellSpan health risk profiling: low risk = 0-8, medium risk = 9-16, high risk = >17												

Selecting an objective quality metric: HbA1c adherence proportions

The metric "Hemoglobin A1c (HbA1c) testing" was selected from the Comprehensive Diabetes Care[7] to evaluate quality of T2DM management and screening. The national committee for quality assurance (NCQA) describes this measure as the percentage of patients with diabetes who received an HbA1c test during the measurement year (excluding hospitalized patients)[8]. To evaluate percentage of adherence in screening versus management of HbA1c lab, the CQMC measure description was modified to include all patients in the denominator. For all group's proportions of adherence, the denominator was defined as "encounters meeting inclusion criteria" and the numerator was defined as "encounters with a linked subsequent completed HbA1c lab order within 6-months".

Using slicer dicer for sub-group analysis: importance of avoiding case-mis adjustments

Using Epic's slicer dicer tool to mine patient encounter data in separate data sessions facilitated the comparison of office and telemedicine encounters (which included both phone and video visits). Results were "sliced" for patients with and without a diagnosis of T2DM (ICD-10 E11* code grouper) to compare management and screening HbA1c lab adherence respectively. Additional data sessions sliced the same population to compare family medicine HbA1c adherence to all other outpatient specialties.

We avoided case-mix adjustments or timeframe selections in our data set since the NQF states that this can impair validity and reliability of results[3,4]. Medcalc's "N-1" Chi-squared calculator[9] was used to detect statistical significance for proportions of HbA1c adherence between sub-groups.

References

1. Alexander GC, Tajanlangit M, Heyward J, Mansour O, Qato DM, Stafford RS. Use and Content of Primary Care Office-Based vs Telemedicine Care Visits During the COVID-19 Pandemic in the US. *JAMA Netw Open*. 2020;3(10):e2021476. doi:10.1001/jamanetworkopen.2020.21476

© 2021 Baughman D et al. *JAMA Network Open.*

2. Eberly LA, Kallan MJ, Julien HM, et al. Patient Characteristics Associated With Telemedicine Access for Primary and Specialty Ambulatory Care During the COVID-19 Pandemic. *JAMA Netw Open*. 2020;3(12):e2031640. doi:10.1001/jamanetworkopen.2020.31640
3. NQF: Measuring Performance. Accessed March 20, 2021. https://www.qualityforum.org/Measuring_Performance/Measuring_Performance.aspx
4. NQF: Quality Positioning System ™. Accessed March 20, 2021. https://www.qualityforum.org/QPS/QPSTool.aspx?m=850&e=1#qpsPageState=%7B%22TabType%22%3A1
5. D'Hoore W, Bouckaert A, Tilquin C. Practical considerations on the use of the Charlson comorbidity index with administrative data bases. *J Clin Epidemiol*. 1996;49(12):1429-1433. doi:10.1016/s0895-4356(96)00271-5
6. Risk Adjustment | CMS. Accessed March 20, 2021. https://www.cms.gov/Medicare/Health-Plans/MedicareAdvtgSpecRateStats/Risk-Adjustors
7. NQF: Accessed March 20, 2021. http://www.qualityforum.org/cqmc/
8. Comprehensive Diabetes Care. NCQA. Accessed March 20, 2021. https://www.ncqa.org/hedis/measures/comprehensive-diabetes-care/
9. Schoonjans F. MedCalc's Comparison of proportions calculator. MedCalc. Accessed March 20, 2021. https://www.medcalc.org/calc/comparison_of_proportions.php

© 2021 Baughman D et al. *JAMA Network Open.*
